# Overexpression of heat shock protein 27 (HSP27) increases gemcitabine sensitivity in pancreatic cancer cells through S-phase arrest and apoptosis

**DOI:** 10.1111/jcmm.12444

**Published:** 2014-10-21

**Authors:** Yang Guo, Andreas Ziesch, Sandra Hocke, Eric Kampmann, Stephanie Ochs, Enrico N De Toni, Burkhard Göke, Eike Gallmeier

**Affiliations:** a Department of Medicine II, Ludwig-Maximilians-UniversityMunich, Germany; b Department of Medicine III, Ludwig-Maximilians-UniversityMunich, Germany; c Department of Gastroenterology, Philipps University MarburgMarburg, Germany

**Keywords:** apoptosis, gemcitabine, HSP27, hyperthermia, pancreatic cancer

## Abstract

We previously established a role for HSP27 as a predictive marker for therapeutic response towards gemcitabine in pancreatic cancer. Here, we investigate the underlying mechanisms of HSP27-mediated gemcitabine sensitivity. Utilizing a pancreatic cancer cell model with stable HSP27 overexpression, cell cycle arrest and apoptosis induction were analysed by flow cytometry, nuclear staining, immunoblotting and mitochondrial staining. Drug sensitivity studies were performed by proliferation assays. Hyperthermia was simulated using mild heat shock at 41.8°C. Upon gemcitabine treatment, HSP27-overexpressing cells displayed an early S-phase arrest subsequently followed by a strongly increased sub-G1 fraction. Apoptosis was characterized by PARP-, CASPASE 3-, CASPASE 8-, CASPASE 9- and BIM- activation along with a mitochondrial membrane potential loss. It was reversible through chemical caspase inhibition. Importantly, gemcitabine sensitivity and PARP cleavage were also elicited by heat shock-induced HSP27 overexpression, although to a smaller extent, in a panel of pancreatic cancer cell lines. Finally, HSP27-overexpressing pancreatic cancer cells displayed an increased sensitivity also towards death receptor-targeting agents, suggesting another pro-apoptotic role of HSP27 along the extrinsic apoptosis pathway. Taken together, in contrast to the well-established anti-apoptotic properties of HSP27 in cancer, our study reveals novel pro-apoptotic functions of HSP27—mediated through both the intrinsic and the extrinsic apoptotic pathways—at least in pancreatic cancer cells. HSP27 could represent a predictive marker of therapeutic response towards specific drug classes in pancreatic cancer and provides a novel molecular rationale for current clinical trials applying the combination of gemcitabine with regional hyperthermia in pancreatic cancer patients.

## Introduction

Pancreatic ductal adenocarcinoma is a highly aggressive cancer and is clinically characterized by early metastatic growth, extensive drug-resistance, and high rates of recurrence 1,2. During the past 10 years, numerous chemotherapeutic and molecularly targeted agents have been evaluated alone or in combination in clinical trials. Despite encouraging recent advances 3,4, gemcitabine—introduced more than 15 years ago—remains the standard therapeutic basis in most clinical settings 5. As a result of the common primary or acquired resistance of pancreatic cancer cells towards gemcitabine 6, predictive markers for chemotherapeutic response are urgently needed 7,8. Various potentially predictive biomarkers have previously been reported, including carbohydrate antigen 19-9 (CA19-9) 9,10, human equilibrative nucleoside transporter-1 (hENT1) 11, deoxycytidine kinase 12, ribonucleotide reductase subunits (RRM1 13 and RRM2 14, Notch3 15, Hu protein antigen R (HuR) 16, microRNA expression 17 or heat shock protein 27 (HSP27). Still, none of these markers has yet proven sufficiently robust to achieve clinical implementation in the gemcitabine-based treatment of pancreatic cancer. Particularly, the role of HSP27 as a predictive marker for gemcitabine sensitivity remained controversial 18–20.

HSP27 possesses chaperone-like activity to prevent aggregation of misfolded proteins. It has mainly been implicated in proteasome-mediated protein degradation, cytoskeleton remodelling and the modulation of cell death pathways 21–24. In cancer, HSP27 appears constitutively expressed at high levels in various tumour entities such as lung 25, gastric 26, prostate 27 and pancreatic cancer 28. In some tumour entities, this HSP27 overexpression appears to be associated with prognosis 29, tumour progression 18,30 or response to treatment 31. Thus, HSP27 has been suggested as a diagnostic, predictive or prognostic marker in various tumour types 31,32. However, the specific functional roles of HSP27 on the molecular level remain insufficiently understood and seem variable depending on tumour type or entity 31,33. This applies particularly to the role of HSP27 as a prognostic or predictive marker in pancreatic cancer, for which on the one hand, HSP27 overexpression was associated with poor prognosis 28,34 and chemoresistance towards gemcitabine in some studies 19,35–37, while on the other hand, it was associated with good prognosis and increased sensitivity towards gemcitabine in others 20.

The aim of this study was to investigate the molecular mechanism underlying the previously observed HSP27-mediated hypersensitivity specifically towards gemcitabine in pancreatic cancer cells 20. To this end, a stable HSP27 overexpression model was applied, focusing on cell cycle modulation and apoptosis induction. This was of interest particularly with regard to HSP27-dependent changes in the balance between pro- and anti-apoptotic molecules along the major apoptosis signalling pathways. In addition, the combined effects of heat shock-mediated HSP27 induction and gemcitabine treatment were assessed in a panel of pancreatic cancer cell lines. Our study identified potential clinical implications of HSP27-dependent gemcitabine sensitivity, providing further support for treatment protocols applying regional hyperthermia in combination with gemcitabine in pancreatic cancer patients.

## Materials and methods

### Cell lines and culture

The cell line PL5 was kindly provided by S.E. Kern (Johns Hopkins University, Baltimore, MD, USA). The PL5-derived stably HSP27-transfected clones (PL5/EV, PL5/hu16, PL5/hu18) have been established and described in detail previously 20. All other cell lines were purchased from the European Collection of Cell Cultures (Sigma-Aldrich, Munich, Germany) or the American Type Culture Collection (LGC Standards, Wesel, Germany), respectively. Early-passage primary human pancreatic cancer cell lines 518, 202, 311, 520, 455, PPC-0039 were derived and short-term propagated in our laboratory from surgical specimens of histologically confirmed pancreatic adenocarcinomas. All cell lines were grown in DMEM supplemented with 10% foetal calf serum, L-glutamine and 1% penicillin/streptomycin (PAA, Coelbe, Germany).

### Reagents

Gemcitabine (Lilly, Bad Homburg, Germany) was dissolved at a stock concentration of 10 mM and stored at room temperature. LBY135 (Novartis, Basel, Switzerland) and tigatuzumab (CS-1008; Daiichi Sankyo, Edison, NJ, USA) at stock concentrations of 50 mg/ml and 10 mg/ml, respectively, were stored at −20 and 4°C. The pan-caspase inhibitor, Z-VAD-FMK (Promega, Mannheim, Germany) was supplied at a stock concentration of 20 mM and stored at −20°C.

### Flow cytometry

Cell cycle distribution and subG1-cell fractions were analysed using a fluorescence-activated cell sorter (FACS) (Accuri C6 Flow Cytometer®; BD Biosciences, Heidelberg, Germany) and CFlow Plus software (BD Biosciences) according to the method by Nicoletti 38. In short, cells were seeded in 12-well plates to reach a confluence of 50–60% the next day. Gemcitabine was then added at concentrations of 0, 6.25, 15 or 25 nM. After 24 or 48 hrs, cells were collected, washed and incubated in staining buffer (0.1% sodium citrate, 0.1% Triton X-100, and 50 μg/ml propidium iodide). Sub-G1 events and cell cycle distribution were measured using CFlow Plus software.

### Hoechst staining

Cells were fixed with 4% paraformaldehyde in PBS for 20 min. followed by washing with PBS. Fixed cells were then stained using Hoechst 33342 (Sigma-Aldrich) at 2.5 μg/ml in PBS for 30 min. and examined using a Zeiss Axiovert 135 TV fluorescence microscope (Carl Zeiss, Jena, Germany).

### Immunoblotting

Cells were lysed and protein extracts boiled and loaded on 10–12% polyacrylamide gels. After electrophoresis, proteins were transferred to PVDF membranes, which were blocked for 1 hr in TBS solution containing 0.1% Tween 20 (TBS-T) and 5% milk powder. Membranes were then incubated using primary antibodies in TBS-T containing 5% milk powder or 5% BSA, respectively, at 4°C overnight. The following first antibodies were used: anti-HSP27 (SPA-803; Stressgen/Enzo, Lörrach, Germany); anti-CASPASE 3, anti-CASPASE 8, anti-CASPASE 9, anti-PARP, anti-BCL-2, anti-BCL-xL, anti-BID, anti-BAX, anti-MCL-1 (all Cell Signaling Technology/New England Biolabs GmbH, Frankfurt am Main, Germany); anti-BAK, anti-BIM (BD Biosciences); anti-CHOP, anti-GRP78 (both Santa Cruz Biotechnology, Heidelberg, Germany). Anti-ß-ACTIN antibody (Sigma-Aldrich) served as loading control. The membranes were washed and stained with either antimouse or anti-rabbit HRP-conjugated antibody (GE Healthcare, Freiburg, Germany). Enhanced chemo-luminescence was elicited using ECL Western Blotting Substrate (Thermo Scientific, Schwerte, Germany). All immunoblotting experiments were performed at least in duplicate and representative results are shown. Protein expression levels were additionally quantified by densitometry for ambiguous blots.

### Cell proliferation assays

The assays were performed over a broad range of concentrations covering 100–0% cell survival. Cells/well of 600–2500 were seeded in 96-well plates to reach confluence on day 6. After settling, the cells were incubated with various drugs at the indicated concentrations. For caspase inhibition experiments, cells were pre-treated using the Z-VAD-FMK pan-caspase inhibitor 39 at 20 μM for 3 hrs and then treated with gemcitabine. Following incubation for 6 days, the cells were washed, lysed in 100 μl H_2_O, and 0.2% SybrGreen (Invitrogen, Darmstadt, Germany) was added. Fluorescence was measured using a CytoFluor 4000 plate reader (Applied Biosystems, Darmstadt, Germany) and growth inhibition calculated as compared to the untreated control samples. At least three independent experiments were performed per agent, with each data point reflecting triplicate wells.

### Evaluation of mitochondrial membrane potential by JC-1 staining

Cells were cultured in poly-l-Lysine-coated (R&D Systems, Wiesbaden-Nordenstadt, Germany) 6-well or 96-well plates for 24 hrs and then treated with gemcitabine at 0, 25 or 50 nM for 6 or 24 hrs, respectively. Next, cells were stained with 5,5′,6,6′-tetrachloro-1,1′,3,3′-tetraethylbenzimidazol-carbocyaniniodide (JC-1) solution (Sigma-Aldrich) at a final concentration of 25 μg/ml for 30 min. Fluorescence was measured employing a CytoFluor 4000 plate reader at 485 nm (excitation) and at 530 and 580 nm (emission). The ratio of green and red fluorescence signals served as surrogate readout for the mitochondrial membrane potential (Δψ_m_) independent of the mitochondrial mass. For fluorescence imaging, cells were grown directly on slides, treated as described above and pictures taken using the Zeiss Axiovert 135 TV fluorescence microscope (Carl Zeiss). For FACS analysis, cells were harvested and analysed using the Accuri C6 Flow Cytometer® (BD Biosciences). Qualitative and quantitative data were recorded through CFlow Plus software (BD Biosciences). Valinomycin (Sigma-Aldrich) or carbonyl cyanide 3-chlorophenylhydrazone (CCCP, Sigma-Aldrich) served as negative controls. All experiments were performed at least in triplicate.

### Experimental heat shock in combination with gemcitabine treatment

After individual determination of the half maximal inhibitory concentration (IC_50_) of gemcitabine for each cell line, the heat shock conditions were individually optimized for each cell line with regard to maximal HSP27 induction, as determined by immunoblotting, along with minimal toxicity, as determined by proliferation assays. After cell seeding and attachment, culture dishes at 60–70% confluence were wrapped with parafilm and immersed in a water bath at 41.8 or 43°C for 15, 30, 60, 90, 120 or 150 min., respectively. Cells subjected to 37°C served as controls. Constant temperature was verified through continuous monitoring. Afterwards, the cells were recovered at 37°C for 24 hrs and harvested for immunoblotting. In another set of experiments, heat shock was performed at 41.8°C for 0, 60 or 90 min., respectively, depending on the respective cell line. Afterwards, cells were incubated with gemcitabine at 0, 25, 50, 100 or 200 nM for 5 days. Cell survival rates were measured using a CytoFluor 4000 plate reader. Experiments were repeated at least in triplicate.

### Statistical analysis

All statistical analyses were performed with IBM SPSS Statistics 21 (SPSS Inc., Chicago, IL, USA). Error bars represent standard error of the mean (SEM) from three experiments. Sensitivity studies were statistically interpreted using a paired Student's *t*-test. *P* < 0.05 were considered statistically significant.

## Results

### Mediation of HSP27-dependent gemcitabine sensitivity through early S-phase arrest and consequent apoptosis

**Fig 1:**
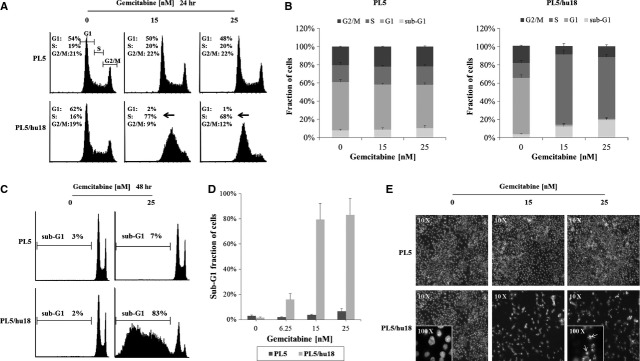
HSP27 overexpression-mediated gemcitabine sensitivity through early S-phase arrest and consequent apoptosis. Flow cytometry comparing cell cycle distribution and sub-G1 cell fractions upon gemcitabine treatment at 0, 15 or 25 nM for 24 or 48 hrs between parental PL5 cells and their HSP27-overexpressing counterparts PL5/hu18: Histograms of representative cell cycle distributions (A and C) and statistical analyses from at least three independent experiments (B and D). Error bars represent SEM. (E) Representative microscopic pictures (magnification, ×10 and ×100) displaying typical morphological features of chromatin condensation and nuclear fragmentation upon gemcitabine at 0, 15 or 25 nM in PL5/hu18 cells (inserts and arrows).

To investigate the mechanism underlying HSP27-mediated gemcitabine sensitivity, we applied flow cytometry to analyse cell cycle distribution and quantify the sub-G1 cell fraction as surrogate marker for apoptosis upon treatment of cells with gemcitabine, comparing parental PL5 cells to their corresponding HSP27-overexpressing counterparts (labelled PL5/hu18). As compared to parental cells, PL5/hu18 cells displayed a strongly increased S-phase fraction (50%) 24 hrs after gemcitabine treatment at 15 or 25 nM (Fig.1A and B). While only a slightly increased sub-G1 fraction (∽15%) was detectable at the time of S-phase arrest (Fig.1B), a strong and dose-dependent increase (∽90%) was observed at 48 hrs in PL5/hu18 cells (Fig.1C and D). Apoptosis indicated by the sub-G1 cell fraction was morphologically validated using nuclear staining. Consistently, increased chromatin condensation and nuclear fragmentation were observed (Fig.1E). Thus, HSP27-dependent gemcitabine sensitivity was mediated first through S-phase arrest and consequently through apoptosis.

### HSP27-dependent cleavage of PARP, CASPASE 3, CASPASE 8 and CASPASE 9 upon gemcitabine

**Fig 2:**
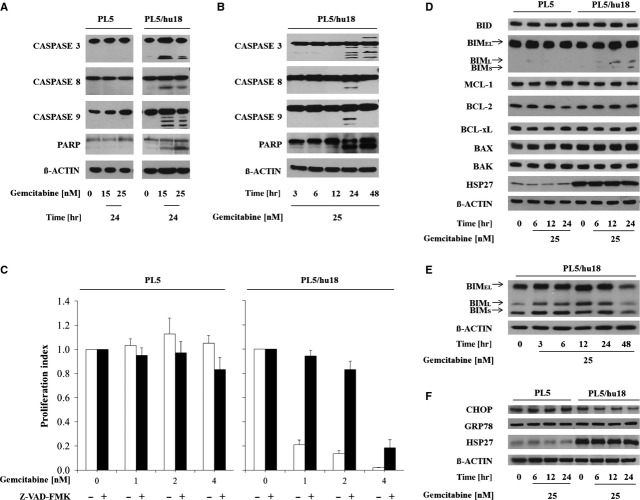
Activation of PARP, caspases and other apoptosis mediators during HSP27-dependent gemcitabine-induced apoptosis. (A and B) Immunoblotting showing cleavage of PARP, CASPASE 3, CASPASE 8 and CASPASE 9 upon gemcitabine treatment in HSP27-overexpressing PL5/hu18 but not parental control cells in a dose-dependent (A) and time-dependent (B) manner. (C) Proliferation assays displaying abrogation of HSP27-dependent gemcitabine sensitivity through pre-incubation with the pan-caspase inhibitor Z-VAD-FMK at 20 μM for 3 hrs. Error bars represent SEM of at least three independent experiments. (D) Immunoblotting assessing expression changes of the indicated pro- or anti-apoptotic molecules in parental PL5 and PL5/hu18 cells upon treatment with gemcitabine at 25 nM at the indicated time-points. ß-ACTIN served as loading control. (E) Immunoblotting displaying time-dependent BIM expression changes in PL5/hu18 cells treated with gemcitabine at 25 nM. (F) Immunoblotting assessing expression changes of CHOP and GRP78 in parental PL5 and PL5/hu18 cells upon treatment with gemcitabine at 25 nM at the indicated time-points. ß-ACTIN served as loading control. Confirmatory HSP27 overexpression in PL5/hu18 cells is additionally depicted.

To confirm and validate HSP27-dependent induction of apoptosis upon gemcitabine treatment, we assessed Poly (ADP-ribose) polymerase (PARP) cleavage as a general apoptosis marker, followed by assessment of cleavage of initiator caspases CASPASE 8 and CASPASE 9 and of the central effector CASPASE 3. A dose- and time-dependent cleavage of PARP and all three caspases was observed virtually exclusively in the HSP27-overexpressing PL5/hu18 but not in parental control cells (Fig.2A and B).

### Caspase inhibitor-induced abrogation of HSP27-dependent gemcitabine sensitivity

To confirm caspase-dependency of HSP27-mediated gemcitabine-induced apoptosis, the caspase inhibitor Z-VAD-FMK 39 was utilized. Consistent with the observed cleavage of CASPASE 3, CASPASE 8 and CASPASE 9 upon gemcitabine treatment, Z-VAD-FMK at 20 μM for 3 hrs virtually completely reversed gemcitabine sensitivity specifically in HSP27-overexpressing PL5/hu18 cells at 1 or 2 nM of gemcitabine. At higher concentrations (4 nM), caspase-independent gemcitabine toxicity was additionally observed (Fig.2C).

### HSP27-dependent activation of BIM upon gemcitabine

To evaluate which apoptotic molecules participated in caspase-dependent apoptosis, we analysed the cellular expression of major pro- and anti-apoptotic mediators along the intrinsic pathway upon gemcitabine treatment at 25 nM. All three isoforms of pro-apoptotic BIM (BIM_EL_, BIM_L_ and BIM_S_) 40 were significantly up-regulated in a time-dependent manner specifically in HSP27-overexpressing PL5/hu18 but not in parental control cells, while no discernible expression changes were detected in any other pro-apoptotic (BID, BAX and BAK) or anti-apoptotic protein (BCL-xL, BCL-2, MCL-1) (Fig.2D and E) tested. As BIM can be activated *via* CCAAT/enhancer-binding protein homologous protein (CHOP) in certain settings, *e.g*. during endoplasmic reticulum (ER) stress-triggered apoptosis 41, we additionally analysed the expression levels of CHOP and of the chaperone GRP78, which represents a central mediator of ER stress 42 upon treatment with gemcitabine. However, no significantly up-regulated expression levels of either protein were detected at 6–24 hrs after treatment in parental PL5 or PL5/hu18 cells (Fig.2F).

### HSP27-dependent mitochondrial membrane potential loss upon gemcitabine

**Fig 3:**
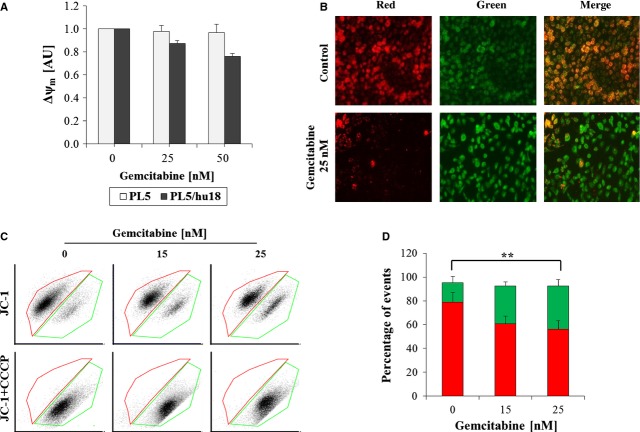
Influence of HSP 27 overexpression on mitochondrial potential upon gemcitabine treatment. (A) Quantitative assessment of dose-dependent mitochondrial membrane potential (Δψ_m_) in PL5/hu18 as compared to parental PL5 control cells 24 hrs after gemcitabine treatment by determination of fluorescence upon JC-1 staining. Error bars represent SEM of three independent experiments. (B) Representative pictures from fluorescent imaging (magnification, ×10) showing JC-1 aggregates (red) and JC-1 monomers (green) in PL5/hu18 cells 24 hrs after treatment with gemcitabine at 25 nM. (C) FACS analyses displaying representative dot blots of dose-dependent Δψ_m_ loss in PL5/hu18 cells (upper panel) and control experiments using CCCP block (lower panel). (D) Statistical analyses of three independent experiments using the Student's t-test (**P < 0.01) with error bars representing SEM.

As BIM is commonly regarded as a central player in the control of mitochondrion-mediated apoptotic processes 43, we next applied three assays using JC-1 staining 44 to analyse mitochondrial membrane depolarization upon HSP27-dependent gemcitabine-induced apoptosis. While under normal conditions, the JC-1 dye concentrates because of the electrochemical potential gradient in the mitochondrial matrix and forms red fluorescent aggregates (JC-1 aggregates), any event that dissipates the mitochondrial membrane potential leads to the prevention of JC-1 accumulation in the mitochondria and JC-1-dispersion throughout the entire cell leading to a shift from red to green fluorescence (JC-1 monomers) 45. Upon gemcitabine at 25 or 50 nM for 24 hrs, Δψ_m_ was quantitatively decreased in HSP27-overexpressing PL5/hu18 but not in parental control cells, as determined by JC-1 fluorescence (Fig.3A). Consistently, fluorescence imaging displayed a strong decrease of JC-1 aggregates in PL5/hu18 cells treated with gemcitabine at 25 nM for 24 hrs (Fig.3B). Similar data were obtained using FACS analysis, in which a clear and dose-dependent shift from JC-1 aggregates to JC-1 monomers was detected in PL5/hu18 cells 24 hrs after treatment with gemcitabine at 15 or 25 nM (Fig.3C and D). This effect was completely blocked by valinomycin (data not shown) or CCCP (Fig.3C), both serving as negative controls. Taken together, these data confirmed the occurrence of mitochondrion-mediated apoptosis specifically in PL5/hu18 cells upon treatment with gemcitabine.

### Heat shock-inducible sensitization of pancreatic cancer cell lines towards gemcitabine

**Fig 4:**
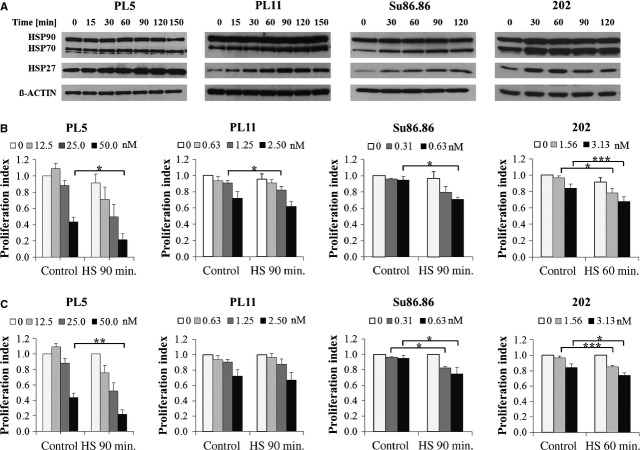
Mild heat shock-mediated HSP27 induction and concomitant sensitization of pancreatic cancer cell lines towards gemcitabine. (A) Immunoblotting displaying the expression levels of HSP27, HSP70 and HSP90 in the indicated established and short-term propagated primary pancreatic cancer cell lines upon heat shock at 41.8°C for 0, 15, 30, 60, 90, 120 or 150 min., respectively. ß-ACTIN served as loading control. (B and C) Proliferation assays comparing the sensitivity towards gemcitabine, with or without prior heat shock (HS) at 41.8°C for the indicated time-points of maximal HSP27 induction, including (B) or excluding (C) the detrimental effects of heat shock alone. Error bars represent SEM of at least three independent experiments. Asterisks mark statistical significance between two samples using the Student's t-test (*P < 0.05 **P < 0.01 ***P < 0.005).

Heat shock induces up-regulation of HSP27 expression in a wide variety of cells including pancreatic cancer cells 20,46–49. We therefore asked, whether heat shock-mediated HSP27 induction would be capable of increasing gemcitabine sensitivity in a similar manner as did engineered HSP27 overexpression in our model, illustrating potential clinical implications of our study 50–53. To test this hypothesis, a panel of established pancreatic cancer cell lines (Capan1, MIA PaCa-2, Panc1, PL5, PL11, Su86.86) as well as short-term propagated, early-passage primary human pancreatic cancer cell lines previously established by us (202, 311, 455, 518, 520, PPC-0039) was screened for heat shock-inducible HSP27 up-regulation along with minimal heat shock-induced toxicity. After identification of suitable cell lines, optimized heat shock conditions with regard to temperature and duration of heat shock were defined. At conditions causing only minimal heat-induced toxicity (41.8°C for 30–120 min., depending on the respective cell line), seven out of twelve lines were HSP27-inducible, which was defined as a HSP27 protein up-regulation of at least 1.5-fold (PL5, PL11, Su86.86, 202, 311, 455, PPC-0039). Of these, four lines were picked for the subsequent experiments according to their favourable growth characteristics in cell culture (PL5, PL11, Su86.86, 202). At the previously determined individual time-points of maximal heat shock-mediated HSP27 induction (Fig.4A), all cell lines displayed significantly increased sensitivity towards gemcitabine (Fig.4B). Similar results were obtained when excluding the slight detrimental effects of mild heat shock alone (*i.e*. when defining the surviving fraction of cells treated with heat shock but without gemcitabine as 100%; Fig.4C). Taken together, mild heat shock enhanced the sensitivity of multiple pancreatic cancer cell lines towards gemcitabine, independent of directly heat-induced detrimental effects. Of note, because of the optimized heat shock conditions used in our experiments, we were able to selectively induce HSP27 without significant concomitant increases of other HSP protein expression levels in two of four cell lines (Fig.4A). As confirmed by densitometry, except for a significant up-regulation of HSP70 in Su86.86 cells (approximately 2.8-fold) and more subtly also in PL11 cells (approximately 1.8-fold), each without concomitant increases of HSP90, no other significant HSP70 or HSP90 expression changes were detected in any of the tested cell lines.

### Activation of PARP during heat shock-induced gemcitabine sensitization

**Fig 5:**
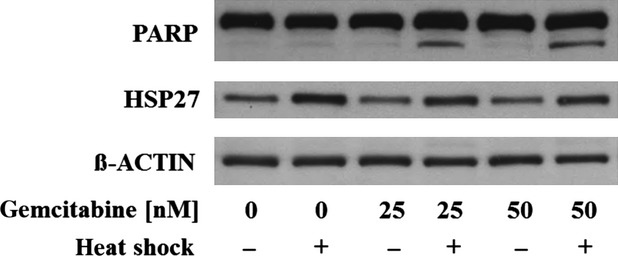
Activation of PARP during heat shock-induced gemcitabine sensitization. Immunoblotting displaying dose-dependent cleavage of PARP in PL5 cells exclusively upon combined treatment with heat shock at 41.8°C for 90 min., followed by gemcitabine at 25 or 50 nM for 24 hrs, but not upon either treatment alone. ß-ACTIN served as loading control.

To gain insight into the potential mechanisms mediating the heat shock-induced gemcitabine sensitization, we analysed PARP as a highly sensitive and early apoptosis marker 54 along with the concomitant cell cycle profiles of PL5 cells, treated with mild heat shock at 41.8°C for 90 min., followed by gemcitabine at 25 or 50 nM, respectively. At 24 and 48 hrs after gemcitabine treatment, no significant changes in cell cycle profiles were detected (data not shown). In contrast, we observed the dose-dependent cleavage of PARP in PL5 cells exclusively after the combined treatment with heat shock and gemcitabine, but not upon either treatment alone (Fig.5), supporting the pro-apoptotic role of HSP27 during heat shock-induced gemcitabine sensitization.

### Impact of HSP27-overexpression on death receptor-targeting agents

**Fig 6:**
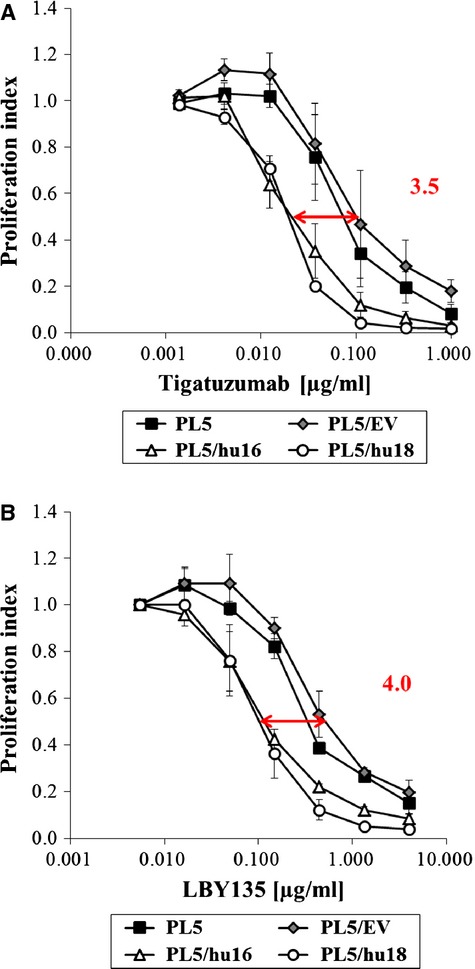
Influence of HSP27 overexpression on the cellular sensitivity towards DR-targeting agents. (A and B) Proliferation assays comparing the sensitivity of parental PL5 and empty vector-transfected (PL5/EV) control cells *versus* two independently derived HSP27-overexpressing cell clones (PL5/hu16, PL5/hu18) towards DR5-agonistic antibodies tigatuzumab (A) or LBY135 (B), respectively at the indicated concentrations. Error bars represent SEM of at least three independent experiments.

As we unexpectedly found HSP27-dependent gemcitabine sensitivity to be partly mediated through CASPASE 8, indicating involvement of the extrinsic apoptosis pathway (Fig.2A and B), we asked whether HSP27 overexpression would accordingly directly influence the cellular sensitivity towards drugs targeting the extrinsic death receptor (DR)-pathway. To test this, parental PL5, empty-vector transfected PL5/EV and two HSP27-overexpressing cell clones (PL5/hu16, PL5/hu18) were treated with tumour necrosis factor-related apoptosis-inducing ligand (TRAIL) receptor-targeting agents currently tested in clinical trials, specifically the anti-DR5 agonistic antibodies tigatuzumab and LBY135. As determined by the IC_50_ ratios, two independently derived HSP27-overexpressing cell clones (PL5/hu16, PL5/hu18) were approximately three to fourfold more sensitive towards these drugs than parental or empty-vector transfected (PL5/EV) control cells, supporting our hypothesis that overexpression of HSP27 not only modulated the cellular sensitivity towards gemcitabine but also towards agents directly targeting the extrinsic DR pathway (Fig.6).

## Discussion

Having previously shown that HSP27 might represent a predictive marker for gemcitabine response in pancreatic cancer, this study served to depict the underlying mechanism of HSP27-mediated gemcitabine sensitivity in pancreatic cancer cells. Here, we demonstrate that gemcitabine treatment caused an early S-phase arrest followed by BIM-, mitochondrion- and caspase-mediated apoptosis specifically in HSP27-overexpressing pancreatic cancer cells. Furthermore, we were able to extend and generalize our data by showing that mild heat shock-mediated HSP27 induction increased the gemcitabine sensitivity in a panel of pancreatic cancer cell lines, although to a smaller extent than did genetically engineered HSP27 overexpression. Finally, our study unexpectedly revealed that HSP27 overexpression sensitized pancreatic cancer cells not only towards gemcitabine but also towards the DR5-targeting agonistic antibodies tigatuzumab and LBY135.

The predominant cell cycle effect observed in our experiments, *i.e*. the early S-phase arrest in HSP27-overexpressing pancreatic cancer cells treated with gemcitabine, is in concordance with the known pharmacological mechanism of action of gemcitabine: The nucleoside analogue belongs to the chemotherapeutic group of antimetabolites, and, after cell entry and phosphorylation, becomes incorporated into DNA. This DNA incorporation causes stalled replication forks during replication and consequently leads to an S-phase checkpoint activation followed by an S-phase arrest 55. In our experiments, this S-phase arrest was subsequently followed by caspase-mediated apoptosis, as illustrated by the activation of the initiator caspases CASPASE 8 and CASPASE 9 and the executioner CASPASE 3 56 and confirmed by the virtually complete abrogation of these effects by a broad caspase inhibitor 39. Thus, the observed gemcitabine-induced pro-apoptotic effects of HSP27 overexpression in pancreatic cancer cells were strictly caspase-dependent in our experiments, whereas the anti-apoptotic properties of HSP27 in other cancers have been reported to be mediated partly by caspase-dependent and partly by caspase-independent mechanisms 57.

HSP27-dependent gemcitabine-induced apoptosis was accompanied by activation of BIM, a pro-apoptotic protein belonging to the BH3-only group of Bcl-2 family members 58, which has just recently been linked to gemcitabine sensitivity in pancreatic cancer 59. Despite BIM being activated *via* CHOP-mediated direct transcriptional induction in certain settings, *e.g*. during ER stress-induced apoptosis 41,60, neither CHOP nor GRP78 42 expression levels were significantly up-regulated upon treatment with gemcitabine in our model, indicating that both CHOP and ER stress did not play a major role in the facilitation of HSP27-dependent gemcitabine sensitization in our experimental setting. In contrast, the fact that BIM is tightly involved in mitochondrion-mediated apoptosis 43 was mechanistically substantiated by our data on gemcitabine-induced mitochondrial membrane potential loss in HSP27-overexpressing pancreatic cancer cells. Cytoskeleton remodelling represents one of the major functions of HSP27 besides the regulation of apoptosis 21,24. Thus, it is tempting to speculate that HSP27-induced cytoskeleton dissociation of BIM and its consecutive mitochondrial translocation—the previously described process through which BIM exerts its pro-apoptotic effects 43,61—constitutes the initiating event of HSP27-dependent gemcitabine-induced apoptosis. These pro-apoptotic effects of HSP27 overexpression probably counteract and potentially outweigh any anti-apoptotic properties of HSP27 under certain circumstances or in certain subsets of tumours. This supports our previously established hypothesis of an important pro-apoptotic role of HSP27 in pancreatic cancer 20, which appears mechanistically clearly distinguishable from the well-established anti-apoptotic properties of HSP27 in a variety of other cancers 18,23.

Experimental heat shock induces HSP27 expression in a wide variety of cell types including pancreatic cancer 20,46–49. Therefore, it appeared mandatory to test whether heat shock-mediated HSP27 induction would be capable of increasing gemcitabine sensitivity in a similar manner as did engineered HSP27 overexpression in pancreatic cancer cells. In fact, we were able to demonstrate that mild experimental heat shock enhanced the sensitivity towards gemcitabine in the majority of cell lines from a panel of established as well as short-term propagated primary pancreatic cancer cell lines, although to a smaller extent than did forced HSP27 overexpression. The effects of combined treatment with heat shock and gemcitabine were accompanied by increased PARP cleavage, indicating that gemcitabine sensitization was attributable at least in part to PARP-mediated apoptosis. Importantly, individual pre-optimization of each cell line facilitated the definition of particularly mild heat shock conditions causing only minimal cellular toxicity, which helped to discriminate the heat shock-induced increase in cell death upon gemcitabine from potential directly heat-induced detrimental effects.

Our data underscore the concept of clinical trials applying treatment protocols including regional hyperthermia in combination with gemcitabine for pancreatic cancer patients, providing a novel molecular mechanism for the clinically established value of this combination 50–53, *i.e*. hyperthermia-mediated HSP27 induction and consecutive increased cellular sensitivity towards gemcitabine. Of note, while many studies concentrate on the role of other HSPs, particularly of HSP70, as critical mediators of the molecular effects observed upon hyperthermia 62, the effects described in our study were likely elicited in a HSP70- and HSP90-independent manner, as can be derived from the optimized heat shock conditions, facilitating HSP27 induction without significant increases of HSP70 in two out of four cell lines and without significant increases of HSP90 in any of the assessed lines. Nevertheless, to definitely dissect the exact role of HSP27 in the process of heat shock-mediated gemcitabine sensitization, the specific downregulation of HSP27, *e.g*. through RNA-interference, will be required. As our own preliminary experiments already revealed that transient siRNA-mediated HSP27 knockdown is insufficient to effectively prevent the acute HSP27 up-regulation upon heat shock (data not shown), future studies will need to apply a stable HSP27 knockdown model using shRNA to definitely address this point.

Unexpectedly, we found that gemcitabine-induced HSP27-dependent apoptosis was mediated partly through CASPASE 8, representing the initiator caspase upon extrinsic DR stimulation 63. This indicated a potential cross-talk between the extrinsic and intrinsic apoptosis pathways. A highly similar extrinsic/intrinsic cross-talk, functionally linking BIM- and mitochondrion-dependent intrinsic apoptotic signalling (both observed in our experiments) to the extrinsic DR cascade, has previously been reported in a different experimental setting 64. Consequently we asked, whether HSP27 overexpression influenced sensitivity not only towards gemcitabine but additionally towards drugs directly targeting the extrinsic DR pathway. In fact, we found that HSP27-overexpressing cells displayed an increased sensitivity towards the DR5-agonistic antibodies tigatuzumab and LBY135, thus potentially identifying a new predictive marker of therapeutic response towards this drug class in pancreatic cancer. In this context, it will be important to test in future studies, whether heat shock-mediated HSP27 induction increased sensitivity not only towards gemcitabine, as shown here but also similarly also towards DR-targeting drugs in pancreatic cancer. Recent studies already support this hypothesis, reporting hyperthermia to enhance apoptosis induced by TRAIL or the DR4-targeting mapatumumab in colon cancer cells *in vitro* and *in vivo* 65,66.

In summary, we identified and mechanistically characterized a novel link between HSP27 expression and gemcitabine sensitivity in pancreatic cancer cells. In contrast to the well-established anti-apoptotic roles of HSP27, our study revealed clearly distinguishable pro-apoptotic functions of HSP27 in certain subsets of cancer cells. This could have direct clinical implications: First, HSP27 might serve as a predictive marker of therapeutic response towards gemcitabine or DR-targeting drugs in pancreatic cancer. Second, our data further substantiate the molecular basis for clinical trials applying combinations of gemcitabine and regional hyperthermia for the treatment of pancreatic cancer 50–53.

## Supporting Information

**Data S1** Response to the reviewers.

## References

[b1] Strobel O, Hartwig W, Hackert T (2013). Re-resection for isolated local recurrence of pancreatic cancer is feasible, safe, and associated with encouraging survival. Ann Surg Oncol.

[b2] Gungor C, Hofmann BT, Wolters-Eisfeld G (2014). Pancreatic cancer. Br J Pharmacol.

[b3] Conroy T, Desseigne F, Ychou M (2011). FOLFIRINOX *versus* gemcitabine for metastatic pancreatic cancer. N Engl J Med.

[b4] Von Hoff DD, Ervin T, Arena FP (2013). Increased survival in pancreatic cancer with nab-paclitaxel plus gemcitabine. N Engl J Med.

[b5] Berlin J, Benson AB (2010). Chemotherapy: gemcitabine remains the standard of care for pancreatic cancer. Nat Rev Clin Oncol.

[b6] Long J, Zhang Y, Yu X (2011). Overcoming drug resistance in pancreatic cancer. Expert Opin Ther Targets.

[b7] Dhayat S, Mardin WA, Mees ST (2011). Epigenetic markers for chemosensitivity and chemoresistance in pancreatic cancer–a review. Int J Cancer.

[b8] Soo RA, Yong WP, Innocenti F (2012). Systemic therapies for pancreatic cancer–the role of pharmacogenetics. Curr Drug Targets.

[b9] Heinemann V, Schermuly MM, Stieber P (1999). CA19-9: a pedictor of response in pancreatic cancer treated with gemcitabine and cisplatin. Anticancer Res.

[b10] Bauer TM, El-Rayes BF, Li X (2013). Carbohydrate antigen 19-9 is a prognostic and predictive biomarker in patients with advanced pancreatic cancer who receive gemcitabine-containing chemotherapy: a pooled analysis of 6 prospective trials. Cancer.

[b11] Giovannetti E, Del Tacca M, Mey V (2006). Transcription analysis of human equilibrative nucleoside transporter-1 predicts survival in pancreas cancer patients treated with gemcitabine. Cancer Res.

[b12] Fujita H, Ohuchida K, Mizumoto K (2010). Gene expression levels as predictive markers of outcome in pancreatic cancer after gemcitabine-based adjuvant chemotherapy. Neoplasia.

[b13] Nakahira S, Nakamori S, Tsujie M (2007). Involvement of ribonucleotide reductase M1 subunit overexpression in gemcitabine resistance of human pancreatic cancer. Int J Cancer.

[b14] Nakano Y, Tanno S, Koizumi K (2007). Gemcitabine chemoresistance and molecular markers associated with gemcitabine transport and metabolism in human pancreatic cancer cells. Br J Cancer.

[b15] Eto K, Kawakami H, Kuwatani M (2013). Human equilibrative nucleoside transporter 1 and Notch3 can predict gemcitabine effects in patients with unresectable pancreatic cancer. Br J Cancer.

[b16] Richards NG, Rittenhouse DW, Freydin B (2010). HuR status is a powerful marker for prognosis and response to gemcitabine-based chemotherapy for resected pancreatic ductal adenocarcinoma patients. Ann Surg.

[b17] Ohuchida K, Mizumoto K, Kayashima T (2011). MicroRNA expression as a predictive marker for gemcitabine response after surgical resection of pancreatic cancer. Ann Surg Oncol.

[b18] Ciocca DR, Arrigo AP, Calderwood SK (2013). Heat shock proteins and heat shock factor 1 in carcinogenesis and tumor development: an update. Arch Toxicol.

[b19] Mori-Iwamoto S, Kuramitsu Y, Ryozawa S (2007). Proteomics finding heat shock protein 27 as a biomarker for resistance of pancreatic cancer cells to gemcitabine. Int J Oncol.

[b20] Schafer C, Seeliger H, Bader DC (2012). Heat shock protein 27 as a prognostic and predictive biomarker in pancreatic ductal adenocarcinoma. J Cell Mol Med.

[b21] Acunzo J, Katsogiannou M, Rocchi P (2012). Small heat shock proteins HSP27 (HspB1), alphaB-crystallin (HspB5) and HSP22 (HspB8) as regulators of cell death. Int J Biochem Cell Biol.

[b22] Ciocca DR, Oesterreich S, Chamness GC (1993). Biological and clinical implications of heat shock protein 27,000 (Hsp27): a review. J Natl Cancer Inst.

[b23] Garrido C, Brunet M, Didelot C (2006). Heat shock proteins 27 and 70: anti-apoptotic proteins with tumorigenic properties. Cell Cycle.

[b24] Wettstein G, Bellaye PS, Micheau O (2012). Small heat shock proteins and the cytoskeleton: an essential interplay for cell integrity?. Int J Biochem Cell Biol.

[b25] Malusecka E, Zborek A, Krzyzowska-Gruca S (2001). Expression of heat shock proteins HSP70 and HSP27 in primary non-small cell lung carcinomas. An immunohistochemical study. Anticancer Res.

[b26] Huang Q, Ye J, Huang Q (2010). Heat shock protein 27 is over-expressed in tumor tissues and increased in sera of patients with gastric adenocarcinoma. Clin Chem Lab Med.

[b27] Rocchi P, So A, Kojima S (2004). Heat shock protein 27 increases after androgen ablation and plays a cytoprotective role in hormone-refractory prostate cancer. Cancer Res.

[b28] Baylot V, Andrieu C, Katsogiannou M (2011). OGX-427 inhibits tumor progression and enhances gemcitabine chemotherapy in pancreatic cancer. Cell Death Dis.

[b29] Zoubeidi A, Gleave M (2012). Small heat shock proteins in cancer therapy and prognosis. Int J Biochem Cell Biol.

[b30] Sherman M, Multhoff G (2007). Heat shock proteins in cancer. Ann N Y Acad Sci.

[b31] Ciocca DR, Calderwood SK (2005). Heat shock proteins in cancer: diagnostic, prognostic, predictive, and treatment implications. Cell Stress Chaperones.

[b32] Bunger S, Laubert T, Roblick UJ (2011). Serum biomarkers for improved diagnostic of pancreatic cancer: a current overview. J Cancer Res Clin Oncol.

[b33] Parcellier A, Schmitt E, Brunet M (2005). Small heat shock proteins HSP27 and alphaB-crystallin: cytoprotective and oncogenic functions. Antioxid Redox Signal.

[b34] Tsiaousidou A, Lambropoulou M, Chatzitheoklitos E (2013). B7H4, HSP27 and DJ-1 molecular markers as prognostic factors in pancreatic cancer. Pancreatology.

[b35] Kuramitsu Y, Wang Y, Taba K (2012). Heat-shock protein 27 plays the key role in gemcitabine-resistance of pancreatic cancer cells. Anticancer Res.

[b36] Liu QH, Zhao CY, Zhang J (2012). Role of heat shock protein 27 in gemcitabine-resistant human pancreatic cancer: comparative proteomic analyses. Mol Med Rep.

[b37] Mori-Iwamoto S, Kuramitsu Y, Ryozawa S (2008). A proteomic profiling of gemcitabine resistance in pancreatic cancer cell lines. Mol Med Rep.

[b38] Nicoletti I, Migliorati G, Pagliacci MC (1991). A rapid and simple method for measuring thymocyte apoptosis by propidium iodide staining and flow cytometry. J Immunol Methods.

[b39] Zhu H, Fearnhead HO, Cohen GM (1995). An ICE-like protease is a common mediator of apoptosis induced by diverse stimuli in human monocytic THP.1 cells. FEBS Lett.

[b40] Li R, Moudgil T, Ross HJ (2005). Apoptosis of non-small-cell lung cancer cell lines after paclitaxel treatment involves the BH3-only proapoptotic protein Bim. Cell Death Differ.

[b41] Puthalakath H, O'Reilly LA, Gunn P (2007). ER stress triggers apoptosis by activating BH3-only protein Bim. Cell.

[b42] Lee AS (2005). The ER chaperone and signaling regulator GRP78/BiP as a monitor of endoplasmic reticulum stress. Methods.

[b43] Tong T, Ji J, Jin S (2005). Gadd45a expression induces Bim dissociation from the cytoskeleton and translocation to mitochondria. Mol Cell Biol.

[b44] Feeney CJ, Pennefather PS, Gyulkhandanyan AV (2003). A cuvette-based fluorometric analysis of mitochondrial membrane potential measured in cultured astrocyte monolayers. J Neurosci Methods.

[b45] Reers M, Smiley ST, Mottola-Hartshorn C (1995). Mitochondrial membrane potential monitored by JC-1 dye. Methods Enzymol.

[b46] Coss RA, Storck CW, Daskalakis C (2003). Intracellular acidification abrogates the heat shock response and compromises survival of human melanoma cells. Mol Cancer Ther.

[b47] Kato K, Ito H, Kamei K (1998). Stimulation of the stress-induced expression of stress proteins by curcumin in cultured cells and in rat tissues *in vivo*. Cell Stress Chaperones.

[b48] Rashmi R, Santhosh Kumar TR, Karunagaran D (2003). Human colon cancer cells differ in their sensitivity to curcumin-induced apoptosis and heat shock protects them by inhibiting the release of apoptosis-inducing factor and caspases. FEBS Lett.

[b49] Tabuchi Y, Takasaki I, Wada S (2008). Genes and genetic networks responsive to mild hyperthermia in human lymphoma U937 cells. Int J Hyperthermia.

[b50] Ishikawa T, Kokura S, Sakamoto N (2012). Phase II trial of combined regional hyperthermia and gemcitabine for locally advanced or metastatic pancreatic cancer. Int J Hyperthermia.

[b51] Maluta S, Schaffer M, Pioli F (2011). Regional hyperthermia combined with chemoradiotherapy in primary or recurrent locally advanced pancreatic cancer: an open-label comparative cohort trial. Strahlenther Onkol.

[b52] Ohguri T, Imada H, Yahara K (2008). Concurrent chemoradiotherapy with gemcitabine plus regional hyperthermia for locally advanced pancreatic carcinoma: initial experience. Radiat Med.

[b53] Tschoep-Lechner KE, Milani V, Berger F (2013). Gemcitabine and cisplatin combined with regional hyperthermia as second-line treatment in patients with gemcitabine-refractory advanced pancreatic cancer. Int J Hyperthermia.

[b54] Simbulan-Rosenthal CM, Rosenthal DS, Iyer S (1999). Involvement of PARP and poly(ADP-ribosyl)ation in the early stages of apoptosis and DNA replication. Mol Cell Biochem.

[b55] Ewald B, Sampath D, Plunkett W (2007). H2AX phosphorylation marks gemcitabine-induced stalled replication forks and their collapse upon S-phase checkpoint abrogation. Mol Cancer Ther.

[b56] Chen M, Wang J (2002). Initiator caspases in apoptosis signaling pathways. Apoptosis.

[b57] Sreedhar AS, Csermely P (2004). Heat shock proteins in the regulation of apoptosis: new strategies in tumor therapy: a comprehensive review. Pharmacol Ther.

[b58] O'Connor L, Strasser A, O'Reilly LA (1998). Bim: a novel member of the Bcl-2 family that promotes apoptosis. EMBO J.

[b59] Yan HJ, Liu WS, Sun WH (2012). miR-17-5p inhibitor enhances chemosensitivity to gemcitabine *via* upregulating Bim expression in pancreatic cancer cells. Dig Dis Sci.

[b60] Kubisch CH, Logsdon CD (2008). Endoplasmic reticulum stress and the pancreatic acinar cell. Expert Rev Gastroenterol Hepatol.

[b61] Puthalakath H, Huang DC, O'Reilly LA (1999). The proapoptotic activity of the Bcl-2 family member Bim is regulated by interaction with the dynein motor complex. Mol Cell.

[b62] Issels RD (2008). Hyperthermia adds to chemotherapy. Eur J Cancer.

[b63] Fulda S, Debatin KM (2006). Extrinsic *versus* intrinsic apoptosis pathways in anticancer chemotherapy. Oncogene.

[b64] Han J, Goldstein LA, Gastman BR (2006). Interrelated roles for Mcl-1 and BIM in regulation of TRAIL-mediated mitochondrial apoptosis. J Biol Chem.

[b65] Song X, Kim HC, Kim SY (2012). Hyperthermia-enhanced TRAIL- and mapatumumab-induced apoptotic death is mediated through mitochondria in human colon cancer cells. J Cell Biochem.

[b66] Alcala MA, Park K, Yoo J (2010). Effect of hyperthermia in combination with TRAIL on the JNK-Bim signal transduction pathway and growth of xenograft tumors. J Cell Biochem.

