# Supplementary material for: Overexpression of heat shock protein 27 (HSP27) increases gemcitabine sensitivity in pancreatic cancer cells through S-phase arrest and apoptosis
Source: J Cell Mol Med. 2014 Oct 21;19(2):340–50. doi: 10.1111/jcmm.12444 (PMC4407596; doi:10.1111/jcmm.12444)
Supplement: Supplementary file 1 [file jcmm0019-0340-sd1.docx]

**Response to the reviewers**

**Reviewer 1**

*This manuscript extends the authors earlier work published in J Cell Mol Med in 2012 (Ref 20 this paper) by presenting more mechanistic studies on the interaction of HSP27 and gemcitabine. Most of the work is carried out with the PL5 cell line stably transfected with HSP27. Cells with higher levels of HSP27 were more sensitive to gemcitabine which was shown to induce cell cycle arrest and apoptosis. Gemcitabine was also shown to decrease the mitochondrial membrane potential in HSP27 overexpressing cells. The second major advance was the use of minimal heat shock to increase HSP27 selectively in 5 cell lines of which 4 showed statistically increased sensitivity to gemcitabine. Overall most of the experiments and results are clearly explained.*

*Major points:*

*1. The authors should use siRNA or shRNA to block the upregulation of HSP27 in response to heat shock and show that the effects on gemcitabine sensitivity are truly due to HSP27 and not other molecules induced by heat shock.*

**Response:** We agree that the combination of RNA-interference technology along with heat shock represents an elegant way to dissect the exact role of HSP27 in heat shock-induced gemcitabine sensitization. We therefore per­formed additional siRNA experiments according to the reviewer’s suggestion. Unfortunately, we experienced significant experimental problems using this approach: On the one hand, HSP27 siRNA transfection along with or after heat shock was inapplicable, as preliminary experiments expectedly confirmed that heat shock-induced HSP27 overexpression occurred early and lasted only transiently (≤ 24h), while siRNA-induced HSP27 depletion occurred only later (with a maximal protein knockdown at 48h to 72h after transfection). On the other hand, siRNA transfection prior to heat shock expectedly and efficiently decreased HSP27 protein expression at 48 and 72 h, but did not sufficiently prevent the acute relative upregulation of “de novo” HSP27 protein upon heat shock **(Fig. R1A)**. Consistently, siRNA-mediated HSP27 knockdown applied prior to heat shock did not cause a relative decrease (but rather a non-significant increase) in the cellular sensitivity towards gemcitabine in our experiments (**Fig. R1B + Fig. R1C**), likely additionally indicating the insufficiency of this multi-variable experimental setup.

Thus, given the above shortcomings of siRNA approaches, we agree with the reviewer that experiments using stable HSP27 knockdown through shRNA technology constitute the consequent next step to dissect the role of HSP27 in heat shock-induced gemcitabine sensitivity, but these elaborate experiments appear to be beyond the scope of this study. Nevertheless, to adequately appreciate the reviewer’s valuable re­com­mendation, we now explicitly mention this limitation of our study in the Discussion section of the manu­script, including the new preliminary siRNA data along with the requirement of additional shRNA experiments.

*2. In many of the figures the labels on the axis are unreadable without going to 200% of normal. A larger font should be used. This is especially a problem in Fig 4.*

**Response:** We accordingly modified the font size of all figures to be readable at the original figure size.

**Figure R1**

***Figure R1. Effects of siRNA-mediated HSP27 downregulation on heat shock-induced HSP27 expression and gem­citabine sensitivity of PL5 cells. (A)*** *Immunoblotting displaying the expression levels of HSP27 at the indicated time-points in control-, HSP27 siRNA- and mock-transfected PL5 cells upon heat shock at 41.8 °C for 90 min. ß-ACTIN served as loading control.* ***(B+C)*** *Pro­li­feration assays comparing the sensitivity towards gemcitabine among control-, HSP27 siRNA- and mock- transfected PL5 cells, with or without prior heat shock, including* ***(B)*** *or excluding* ***(C)*** *the detrimental effects of heat-shock alone. Error bars represent SEM of at least three independent experiments.*

**Reviewer 2**

*The authors report that overexpression of heat shock protein 27 (HSP27) increases Gemcitabine sensitivity in pancreatic cancer cells. Drug sensitivity was explored in pancreatic cancer cells with stable HSP27 (wild-type) overexpression and in heat shocked cells and they found that the effect was due to s-phase arrest and by inducing novel pro-apoptotic functions of HSP27 mediated through both the intrinsic and the extrinsic apoptotic pathways. This is an important and controversial issue since it is well known that HSP27 is involved in anti-apoptotic pathways and in drug resistance.*

*This work complements another reported previously by the same authors (where HSP27 was down-regulated), taken together the materials and methods and the results support the conclusions. Here they explored more the mechanistic effects of HSP27 on apoptosis. The results are well presented.*

*Observations:*

*1. The authors show Bim expression changes after drug treatment (in relation to HSP27) (Fig. 2). This is an important observation that needs expansion, Bim is a CHOP-regulated proapoptotic protein, in relation to TRAIL-induced apoptosis. These mechanisms have not been explored and are important to understand the role of Gemcitabine in relation to HSP27.*

**Response:** We thank the reviewer for suggesting CCAAT/enhancer-binding protein homologous protein (CHOP) as a potential regulator of BIM upregulation upon gemcitabine treatment in HSP27-overexpressing cells. We tested this hypothesis through assessment of CHOP expression levels in parental and HSP27-overexpressing PL5/hu18 cells upon treatment with gemcitabine. Because BIM can also become activated via CHOP-mediated direct transcriptional induction during endoplasmic reticulum (ER) stress-induced apoptosis [1,2], we additionally assessed the expression levels of a central mediator of ER stress, i.e. the chaperone GRP 78 [3]. However, neither CHOP nor GRP78 expression levels were significantly upregulated upon gemcitabine in parental PL5 or PL5/hu18 cells at 6, 12 or 24 h after treatment (**Fig. R2)**, indicating that in our experimental model CHOP did not act as an upstream regulator of BIM and ER stress did not play a major role in the facilitation of HSP27-dependent gemcitabine sensitization.

We accordingly modified the Results and Discus­sion sections of the manuscript and added a new figure panel (Fig. 2F). Never­the­less, further studies on the regulation of BIM induction are warranted in order to obtain a clearer picture of the apoptotic processes facilitating HSP27-dependent gemcitabine sensitization.

*2. The heat shock treatment is increasing other HSPs (in addition to HSP27), this can be noted in the western blots (Fig. 4) starting from line PL11, the authors need to add a densitometry to the western blots. They should relate these increments with the possible effects of Gemcitabine.*

**Response:** We agree with the reviewer that the mere presentation of HSP27, HSP70 and HSP90 protein ex­pres­sion levels in figure 4 along with the statement that “due to the optimized heat shock conditions”, HSP27 was in­duced “without significant concomitant increase of HSP70 or HSP90 expression levels in most cell lines” is too vague and should be more precisely defined. We therefore performed densitometry analyses for all im­mu­no­blots in Fig. 4 (**Fig. R3**). In fact, the reviewer correctly discerned that in addition to the HSP27 changes, HSP70 (but not HSP90) was significantly upregulated in Su86.86 cells (approximately 2.8-fold) upon heat shock. More subtly, HSP70 was also upregulated in PL11 cells (approximately 1.8-fold). No other signi­fi­cant changes (defined as a ≥ 1.5-fold upregulation) were found regarding either HSP70 or HSP90 in any of the cell lines.

However, the new densitometric analyses also unexpectedly revealed that we overestimated the upregulation of HSP27 in 518 cells from the blots, which was densitometrically below 1.5-fold. As heat shock-inducible HSP27 upregulation was originally used as the inclusion criterion during the cell line selection process (see Results section, paragraph “*Heat shock-inducible sensitization of pancreatic cancer cell lines towards gemci­ta­bine*“), we now con­se­quent­ly removed 518 cells from our study. In fact, the comparatively small HSP27 up­re­gula­tion upon heat shock in 518 cells as compared to the effects on the other cell lines might explain why 518 was the only tested cell line that did not reach statistical significance in our gemcitabine sensitivity assays.

We there­fore thank the reviewer for his important criticism and accordingly corrected the respective sections of the manuscript, including the exact data from the densitometric analyses. We believe that these corrections signifi­cantly improved our manuscript. Nevertheless, experiments applying the specific down­regu­la­tion of HSP27, e.g. through shRNA-mediated RNA-interference, will eventually be required to definitely dissect the exact role of HSP27 versus HSP70 and HSP90 in the process of heat shock-mediated gemcitabine sensitization. In this regard, please also refer to Reviewer 1 point 1.

*Minor observations:
Page 8, line 29: … mitochondrial mass; For … (should be: … mitochondrial mass. For ...).*

*Page 8, line 34: … (Carl Zeiss); For FACS … (should be: … (Carl Zeiss). For FACS …).*

**Response:** We accordingly modified the manuscript.

**Figure R2**

***Figure R2. CHOP and GRP78 expression during HSP27-dependent gemcitabine sensitization.*** *Immuno­blot­ting assessing expression changes of CHOP and GRP78 in parental PL5 and PL5/hu18 cells upon treatment with gemcitabine at 25 nM at the indicated time points. ß-ACTIN served as the loading control. Confirmatory HSP27 over­ex­pres­sion in PL5/hu18 cells is additionally depicted. Experiments were performed at least in duplicate and representative results are shown.*

**Figure R3**

***Figure R3. Densitometric analyses of heat shock-induced HSP expression.*** *Histograms displaying the relative expression levels of HSP27, HSP70, and HSP90 in relation to the corresponding expression levels of ß-ACTIN for the indicated cell lines upon heat shock. HSP/ß-ACTIN-ratios of the untreated cells were defined as 1.*

**Reviewer 3**

*Guo et al. demonstrate that overexpression of HSP27 not only increases but actually facilitates Gemcitabine-induced apoptosis. Several methods are used to show that HSP27 overexpression dominates in this process. In a number of cell lines a correlation is found between HSP27 expression, induced by mild hyperthermia, and cell proliferation. Finally, the authors argue that the effect of HSP27 has a broader effect through distinct death receptor pathways.
The study is well performed. The authors mention the insight in mechanisms behind the role of HSP27 and indeed try to give some of that. However, they show only some processes which seem to be involved when HSP27 is overexpressed. At that moment we are looking at a rather black and white picture. Overexpression of HSP-27 appears crucial. When shifting to cell lines with intrinsic differences in HSP27 expression and induction of HSP27 expression by higher temperatures the results are less clear and dramatic. Here the authors show limited data. The forced expression of HSP27 is good to start with, but the cell lines and heating of the cells are more important. It is of interest to see what is going on in these cells and at that setting. This next to the effect HSP27 has on processes preventing apoptosis which the author speculate on but did not study.*

**Response:** We agree with the reviewer that it is important to not only implicitly assume but explicitly analyze apoptosis versus cell cycle arrest as the predominant mechanism of heat shock-induced gemcitabine sensitization. Therefore, we additionally analyzed PARP as a highly sensitive and early apoptosis marker [4] along with the concomitant cell cycle profiles of PL5 cells treated with mild heat shock at 41.8 °C, followed by gemcitabine at 25 or 50 nM, respectively. At 24 and 48 h after gemcitabine treatment, no significant changes in cell cycle profiles were detected (data not shown). In contrast, we observed the dose-dependent cleavage of PARP in PL5 cells exclusively upon the combined treatment with heat shock and gemcitabine, but not upon either treatment alone (**Fig. R4**). Our data thus strongly support a pro-apoptotic role for HSP27 as mechanistic explanation for the heat shock-induced gemcitabine sensitization.

We accordingly modified the respective sections of the manuscript and now provide a new figure 5. We thank the reviewer for identifying the absence of mechanistic studies during heat shock-induced gemcitabine sensitization, a crucial mistake that has now been corrected. We believe that the newly obtained data significantly improved our manuscript.

**Figure R4**

***Figure R4. Activation of PARP during heat shock-induced gemcitabine sensitization.*** *Immunoblotting displaying dose-dependent cleavage of PARP in PL5 cells exclusively upon combined treatment with heat shock at 41.8 °C for 90 min., followed by gemcitabine at 25 or 50 nM for 24 h, but not upon either treatment alone. ß-ACTIN served as loading control.*

**References cited in this response**

1.	**Kubisch CH, Logsdon CD.** Endoplasmic reticulum stress and the pancreatic acinar cell. *Expert Rev Gastroenterol Hepatol*. 2008; 2: 249-60.

2.	**Puthalakath H, O'Reilly LA, Gunn P, et al.** ER stress triggers apoptosis by activating BH3-only protein Bim. *Cell*. 2007; 129: 1337-49.

3.	**Lee AS.** The ER chaperone and signaling regulator GRP78/BiP as a monitor of endoplasmic reticulum stress. *Methods*. 2005; 35: 373-81.

4.	**Simbulan-Rosenthal CM, Rosenthal DS, Iyer S, et al.** Involvement of PARP and poly(ADP-ribosyl)ation in the early stages of apoptosis and DNA replication. *Mol Cell Biochem*. 1999; 193: 137-48.
